# Diabetische Neuropathie und diabetischer Fuß (Update 2023)

**DOI:** 10.1007/s00508-023-02167-7

**Published:** 2023-04-20

**Authors:** Gerd Köhler, Marlies Eichner, Heidemarie Abrahamian, Markus Kofler, Wolfgang Sturm, Anja Menzel

**Affiliations:** 1 Klinische Abteilung für Endokrinologie und Diabetologie, Medizinische Universität Graz, Graz, Österreich; 2 Rehabilitationszentrum Aflenz für Stoffwechselerkrankungen mit Schwerpunkt Diabetes mellitus und hochgradige Adipositas, Aflenz, Österreich; 3 Klinik Hietzing, Wien, Österreich; 4 Internistische & Endokrinologische Ordination, Wien, Österreich; 5 Abteilung für Neurologie, Landeskrankenhaus Hochzirl, Hochzirl-Natters, Österreich; 6 Universitätsklinik für Innere Medizin I Innsbruck, Innsbruck, Österreich; 7 Innere Medizin, Endokrinologie und Diabetologie, Deutschlandsberg, Österreich

**Keywords:** Diabetische Neuropathie, Diabetischer Fuß, Diabetic neuropathy, Diabetic foot

## Abstract

Der Begriff der diabetischen Neuropathie ist eine Sammelbezeichnung für Erkrankungen des peripheren Nervensystems die als Spätkomplikation des Diabetes mellitus auftreten.

Die Leitlinienempfehlungen beschreiben die klinischen Symptome und diagnostischen Möglichkeiten, sowie die Therapiemaßnahmen insbesondere bei der schmerzhaften Form der sensomotorischen Neuropathie, einschließlich der komplexen Problematik des diabetischen Fußes.

## Abstract (en)

These are the guidelines for diagnosis and treatment of diabetic neuropathy and diabetic foot.

The position statement summarizes characteristic clinical symptoms and techniques for diagnostic assessment of diabetic neuropathy, including the complex situation of the diabetic foot syndrome. Recommendations for the therapeutic management of diabetic neuropathy, especially for the control of pain in sensorimotor neuropathy, are provided. The needs to prevent and treat diabetic foot syndrome are summarized.

## Diabetische Neuropathie

Unter dem Begriff der diabetischen Neuropathie werden Störungen der peripheren sensomotorischen und autonomen Nervenfunktion zusammengefasst, die in Folge des Diabetes mellitus auftreten und mit vielfältigen klinischen Symptomen einhergehen [1–4]. Typische Symptome der diabetischen Polyneuropathie finden sich bei bis zu 50 % aller Menschen mit Diabetes mellitus, insbesondere bei gezielter Befragung [3–6]. Rund 30–50 % der Menschen mit Diabetes und Neuropathie leiden im Laufe Ihrer Diabeteserkrankung an neuropathischen Schmerzen [2]. Anzeichen einer diabetischen Neuropathie können bereits bei Patienten mit beeinträchtigter Glukosetoleranz bestehen. In der MONICA/KORA-Studie betrug die Prävalenz der Neuropathie rund 30 % bei manifestem Diabetes und 13 % bei Patienten mit gestörter Glukosetoleranz [5]. Durch eingehendere diagnostische Maßnahmen, wie Prüfung der Sehnenreflexe, der Vibrationsempfindung, der Sensibilität, sowie Schmerz- und Temperaturdiskriminierung, werden die Fehlfunktionen und Defizite in Folge einer Neuropathie klinisch gezielt erfasst. Diese Untersuchungen ermöglichen auch die Diagnose einer symptomarmen und schmerzlosen Neuropathie. Die Prävalenz der diabetischen Neuropathie erhöht sich dadurch auf bis zu 60 % der Patienten mit einem manifesten Diabetes mellitus [3, 7, 8] Symptomatische periphere Neuropathieformen werden grundsätzlich häufiger diagnostiziert als eine autonome diabetische Neuropathie.

Die diabetische Neuropathie korreliert mit dem Lebensalter der Patienten, der Diabetesdauer, der glykämischen Kontrolle, dem Tabakkonsum, sowie dem Auftreten weiterer mikroangiopathischer Spätkomplikationen [6, 9, 10]. In die Entwicklung der diabetischen Neuropathie sind komplexe pathophysiologische Mechanismen involviert, die großteils in Folge der Hyperglykämie auftreten [11, 12]. Dazu zählen die Sorbitolakkumulation bei gesteigerter Aldosereduktasereaktion [13]. oxidativer Stress und eine Störung der Blutversorgung über die Vasa nervorum [14]. Funktionsstörungen von Struktur- und Funktionsproteinen durch die nichtenzymatische Glykierung, Störungen im Metabolismus der n‑6-essentiellen Fettsäuren und Prostaglandine mit Änderung der Struktur der Nervenmembranen, sowie ein Mangel an neurotrophen Faktoren und immunologische Mechanismen mit Bildung von Antikörpern gegen neurale Strukturen [12]. Differentialdiagnostisch müssen andere Formen der Neuropathie ausgeschlossen werden, wie die alkoholtoxische Neuropathie, ein Vitamin B12 Mangel, Infektionen (Neuroborreliose, HIV-Infektion), chronisch inflammatorische demyelinisierende Polyneuropathie, Paraproteinämien, Amyloidose und ein paraneoplastisches Geschehen, sowie die Neuropathie in Folge einer Zytostatikatherapie [1]. Von Relevanz ist, dass Metformin zu einer Reduktion der Resorption von Vitamin B12 und in Folge zu einer dadurch bedingten Neuropathie führen kann. Bei Therapie mit Metformin und vorliegender Neuropathie sollten deswegen die Vitamin B12 Spiegel kontrolliert und ggf. substituiert werden [15–17].

## Klinisches Erscheinungsbild der diabetischen Neuropathie

### Distale symmetrische sensomotorische Neuropathie

Die distale symmetrische sensible Neuropathie stellt mit bis zu 70 % die klinisch häufigste Manifestationsform dar [1, 3].

Betroffene berichten typischerweise über Taubheitsgefühl, Parästhesien und/oder Schmerzen an den unteren und oberen Extremitäten. Die Beschwerden breiten sich strumpf- bzw. handschuhförmig von distal nach proximal aus, die Schmerzcharakteristik wird als brennend, bohrend und krampfartig beschrieben („burning feet“), und häufig zeigt sich eine Zunahme während der Nachtstunden.

Klinisch finden sich abgeschwächte oder fehlende Eigenreflexe, Sensibilitätsstörungen, ein herabgesetztes Vibrationsempfinden (Pallhypästhesie) und ein gestörtes Temperaturempfinden (Thermhypästhesie). Ausgeprägte Tiefensensibilitätsstörungen können zu einer sensorischen Ataxie führen. Verzögerungen der Nervenleitgeschwindigkeit und eine Amplitudenreduktion der Nervenaktionspotentiale werden durch elektrophysiologische Untersuchungen erfasst.

Die schmerzhafte diabetische Neuropathie beruht vorwiegend auf Veränderungen der schmerzleitenden dünn-myelinisierten Nervenfasern. Die seltene akute schmerzhafte Neuropathie kann bei Therapieintensivierung eines schlecht eingestellten Diabetes auftreten. Bei einem zusätzlichen Befall der motorischen Fasern finden sich von distal nach proximal fortschreitende Paresen.

### Mononeuropathie

Sowohl Hirnnerven wie auch periphere Nerven können im Rahmen einer Mononeuropathie betroffen sein, mit einem variablen Ausmaß von leichter bis kompletter Parese betroffener Muskeln.. Die vorwiegend bei älteren Patienten zu beobachtende diabetische Ophthalmoplegie beruht auf Ausfällen im Bereich des 3., 4. und 6. Hirnnerven, führt zu Doppelbildern und orbitalen Schmerzen. Diese Form der Neuropathie zeigt jedoch eine günstige Prognose mit Reversibilität innerhalb von 4–6 Wochen. Periphere Ausfälle werden im Bereich des N. medianus und N. peronaeus beobachtet. Auch das Risiko der Entwicklung eines Kompressionssyndroms, insbesondere eines Karpaltunnelsyndroms, ist bei Menschen mit Diabetes erhöht.

### Diabetische Radikulopathie

Diese Form der Neuropathie betrifft die segmentalen thorakalen Spinalnerven. Klinisch finden sich ein- oder doppelseitige gürtelförmige Schmerzen thorakal oder abdominal, Paresen im Bereich der Abdominalmuskulatur und Sensibilitätsausfälle.

### Diabetische Amyotrophie

Die diabetische Amyotrophie ist eine eher seltene Form der diabetischen Neuropathie und tritt vor allem bei Diabetes mellitus Typ 2 und im fortgeschrittenen Lebensalter auf. Im Rahmen einer unilateralen schmerzhaften Neuropathie kann dabei sowohl der lumbosakrale Bereich wie auch der Plexus brachialis betroffen sein. Die Patienten berichten über Schmerzen und deutliche Funktionseinschränkungen. Typisch sind Probleme beim Aufstehen aus dem Sitzen aufgrund rasch progredienter atrophierender Paresen der Oberschenkelmuskulatur. Differentialdiagnostisch muss diese Form deshalb von orthopädischen Erkrankungen abgegrenzt werden. Die Prognose der diabetischen Amyotrophie ist günstig.

### Autonome Polyneuropathie

Grundsätzlich kann die autonome Neuropathie alle Organsysteme betreffen. Klinisch bedeutsam sind die gestörte Hypoglykämiewahrnehmung, das Fehlen von Schmerzen bei myokardialer Ischämie (stummer Myokardinfarkt), die Ruhetachykardie und orthostatische Hypotonie, sowie die gestörte Magenentleerung mit entsprechend schwieriger glykämischer Kontrolle [1, 3, 18, 19].

Das Mortalitätsrisiko für Menschen mit Diabetes mit reduzierter Herzfrequenzvariabilität oder symptomatischer kardiovaskulärer autonomer Neuropathie ist für einen Zeitraum von 5–10 Jahren um das bis zu 5‑fache gesteigert [8]. Für Betroffene besonders belastend sind urologische Manifestationen der autonomen Polyneuropathie, wie die Cystopathie und die erektile Dysfunktion [18, 19]. Die diabetische Cystopathie mit einer Störung der Blasenentleerung kann Anlass für wiederholte Infekte sein, die aufgrund der Sensibilitätsstörungen kaum oder nicht wahrgenommen werden.

## Diagnostik der sensomotorischen Neuropathie

Alle Menschen mit Diabetes müssen regelmäßig auf das Vorliegen einer diabetischen sensomotorischen Neuropathie gescreent werden [1]. Die Erstuntersuchung dazu sollte bei Typ 2 Diabetes zum Zeitpunkt der Diagnosestellung und bei Typ 1 Diabetes nach einer Diabetesdauer von 5 Jahren erfolgen. Regelmäßige Kontrollen sind in jährlichen Intervallen empfohlen [1].

### Anamnese

Anamnestische Daten, die für das Vorliegen einer diabetischen sensomotorischen Neuropathie sprechen, sind symmetrische Parästhesien und/oder Schmerzen an der unteren und seltener an den oberen Extremitäten, die vorwiegend in Ruhe und nachts auftreten.

### Neurologischer Basisstatus

Der optimale klinisch-neurologische Status beinhaltet die Messung des Vibrationsempfindens mittels Rydel-Seiffer Stimmgabeltest zur Erfassung der Pallästhesie, die Beurteilung des Berührungsempfinden durch das Semmes Weinstein Monofilament, die Reflexprüfung, Spitz-Stumpf-Diskriminierung, Temperaturdiskriminierung und Beurteilung des Lagesinnes [1, 20, 21]. Die diagnostischen Maßnahmen werden standardisiert in verschiedenen Scores zur Beurteilung des Schweregrades der diabetischen Neuropathie angeführt [1, 9, 22, 23]. Zu den Diagnosekriterien zählen ein Neuropathie-Defizit-Score von 6–8 oder ein Neuropathie-Defizit-Score von 3–5 mit einem Neuropathie-Symptomen-Score von 4–6. Die Anwendung einer standardisierten Schmerzskala sollte zum vergleichenden Monitoring und zur Überprüfung der Wirkung der therapeutischen Maßnahmen eingesetzt werden.

### Neurophysiologische Diagnostik

Die neurophysiologische Untersuchung stellt den Goldstandard in der Diagnostik einer diabetischen Neuropathie dar [2]. Die elektrophysiologische Abklärung ermöglicht dabei die Darstellung unterschiedlicher Subformen der diabetischen Neuropathie. Eine gemischte sensomotorische und autonome Neuropathie findet sich bei rund 70 % der Patienten, eine sensible Neuropathieform bei rund 30 %. Die sensible Neuropathie wird in 3 Subgruppen unterteilt, wobei 2a die dick-myelinisierten Fasern betrifft, 2b die dünn-myelinisierten Fasern und 2c gemischte Fasern. Eine rein motorische oder rein autonome Neuropathie findet sich nur in jeweils < 1 % der Fälle [24].

Die früheste elektroneurographische Veränderung ist eine Amplitudenabnahme des sensorischen Aktionspotentials des N. suralis (< 6 µV). Weitere sensitive Parameter für eine diabetische Neuropathie sind die sensible Nervenleitgeschwindigkeit des N. suralis und die motorische Nervenleitgeschwindigkeit des N. peronaeus. Die eher milde Verlangsamung der Nervenleitgeschwindigkeit und die Amplitudenabnahme der sensiblen und motorischen Potentiale ist auf einen Axonverlust zurückzuführen. Leitungsblöcke sind an sich untypisch mit Ausnahme von Nervenkompressionssyndromen. Vermehrte temporale Dispersion kommt typischerweise an Engpässen und bei fortgeschrittener Neuropathie vor. An den oberen Extremitäten empfiehlt sich die Untersuchung des N. radialis, der nicht durch Engpässe im Karpaltunnel bzw. Sulcus n. ulnaris beeinträchtigt ist.

Diabetische lumbosakrale Plexopathie bzw. thorakale Radikulopathie sind elektromyographisch durch fokale Denervierung im entsprechenden Myotom gekennzeichnet.

## Diagnostik der autonomen Neuropathie

Ein Screening hinsichtlich einer autonomen Neuropathie wird international im Regelfall aufgrund der geringen zur Verfügung stehenden therapeutischen Maßnahmen nicht durchgeführt, obwohl entsprechende Methoden zur Verfügung stehen würden.

Als einfache klinische Hinweise auf das Vorliegen einer autonomen diabetischen Neuropathie gelten eine Verminderung der Variabilität der Herzfrequenz im Orthostaseversuch und bei Inspiration, sowie ein deutlicher Blutdruckabfall im Aufstehversuch (> 30 mm Hg) [18, 19].

Die *neurovegetative Testung* dient der Erfassung einer isolierten oder begleitenden autonomen Neuropathie. Dazu zählen kardiovaskuläre Parameter (RR-Intervall-Varianz in Ruhe, während forcierter Atmung und während eines Valsalva-Manövers; Kipptischuntersuchung mit Blutdruckmessung im Liegen, Stehen, während Valsalva-Manöver und isometrischer Anspannung; Spektralanalyse), sowie Messung der sympathischen Hautantwort.

Cutane Silent Periods sind einfach zu untersuchen, objektivieren die Funktion von dünn-myelinisierten A‑delta-Fasern [25], sind aber in internationalen Leitlinien noch nicht enthalten [26].

Als Goldstandard in der Abklärung einer „Small-Fiber-Neuropathie“ gilt die Hautstanzbiopsie. Eine neuere nicht-invasive Technik stellt die korneale konfokale Mikroskopie zur Bestimmung der Innervationsdichte, -länge und -verzweigung in der Kornea dar [27, 28]. Beide Methoden besitzen aber im Rahmen eines Diabetes mellitus keine diagnostische Relevanz, es sei denn, eine Neuropathie anderer Ätiologie müsste abgegrenzt werden.

## Psychologische Aspekte der diabetischen Neuropathie und des diabetischen Fußsyndroms

Chronischer psychosozialer Stress, interpersonelle Probleme, mangelnde soziale Ressourcen und nicht geglückte emotionale und/oder kognitive Krankheitsbewältigung können sich negativ auf den emotionalen Zustand auswirken und zu einer Reduktion der gesundheitsbezogenen Lebensqualität führen [29]. Bei Persistenz eines negativen emotionalen Zustandes (Distress, Depression, Angststörung, etc.) über einen längeren Zeitraum kann es zu ungünstigen Auswirkungen auf den Verlauf einer chronischen Erkrankung wie Diabetes mellitus kommen. Das nachteilige Wechselspiel zwischen negativen emotionalen Zustand und Glykämie, sowie Auftreten von Spätschäden des Diabetes mellitus ist in der rezenten Literatur gut dokumentiert [29–31]. In der von der deutschen Diabetesgesellschaft aktuell publizierten Leitlinie „Diabetisches Fußsyndrom“, werden unter den Risikofaktoren für die Entstehung des diabetischen Fußsyndroms psychosoziale Faktoren wie Depression, Vernachlässigung der Selbstfürsorge, Mangel an sozialer Unterstützung und soziale Isolation sowie psychosozialer Stress im weiteren Sinn als kausale Faktoren genannt [31]. Die Aktivierung des neuroendokrinen Stresssystems mit erhöhten Spiegeln von Cortisol, ACTH und CRH, sowie Katecholaminen mit ungünstigen Auswirkungen auf das Immunsystem spielen dabei eine wichtige Rolle [30].

Die diabetische periphere Neuropathie (DPN) kann mit starken Schmerzen verbunden sein, die sich nachteilig auf die Lebensqualität und die sozialen Funktionen von Individuen auswirken, sowie die Manifestation von affektiven Störungen begünstigen können. In einer rezenten internationalen Studie INTERPRET-DD (International Prevalence and Treatment of Diabetes and Depression), wurden 2733 Patienten mit Typ 2 Diabetes mellitus, Alter 18–65 Jahre, Diabetesdauer 8,8 Jahre aus 14 Ländern für die Erhebung der DPN eingeschlossen. Die Gesamtprävalenz von DPN in der Studienpopulation betrug 26,71 %. Die multivariate Analyse ergab, dass die Dauer von Diabetes (OR: 1,08 pro 1‑Jahres-Anstieg, 95 % CI: 1,06–1,09), schlechte glykämische Kontrolle (OR: 1,11 pro 1 % Anstieg von HbA1c, 95 % CI: 1,05–1,18), Bluthochdruck in der Anamnese (OR: 1,58; 95 % KI: 1,18–2,12), Herz-Kreislauf-Erkrankungen (OR: 2,07; 95 % KI: 1,55–2,78) und depressive Symptome (OR: 1,92; 95 % KI: 1,43–2,58) unabhängig und positiv mit dem Risiko von DPN assoziiert waren. Kardiovaskuläre Erkrankungen sowie Symptome der Depression wiesen in dieser Studie den stärksten Zusammenhang mit DPN auf, wobei die Kausalität nicht zugeordnet werden konnte [32, 33]. In der jüngst publizierten kanadischen Studie „Evaluation of Diabetes Treatment“ wurde der signifikante Zusammenhang zwischen ausgeprägten Symptomen der Depression und Risiko für DPN bestätigt, wobei auch für Alkoholkonsum ein hohes Risiko gefunden wurde [34].

Die Berücksichtigung psychosozialer Faktoren ist sowohl bei der Diagnostik der diabetischen Neuropathie und des diabetischen Fußsyndroms als auch im multidisziplinären Therapieansatz von großer Bedeutung [29, 31, 33–35].

## Therapie der diabetischen Polyneuropathie (PNP)

### Kausale Therapie

#### Optimierung der glykämischen Kontrolle

Eine hinreichende Evidenz bezüglich des Einflusses einzelner Risikofaktoren für die Entwicklung und Progression einer diabetischen Polyneuropathie besteht zum aktuellen Zeitpunkt für die meisten dieser Risikofaktoren nicht. Neben den multiplen klinischen Erscheinungsformen der diabetischen Polyneuropathie sind auch unterschiedliche Studiendesigns der entsprechenden Untersuchungen zu Pathogenese und Progression Gründe für diesen Umstand. Alleine für eine normnahe glykämische Kontrolle bei Diabetes mellitus Typ 1 konnte ein verbessertes Ergebnis für die distale symmetrischen Polyneuropathie (DSPN) sowie die kardiovaskuläre autonomen Neuropathie (CAN) gezeigt werden [1–6]. Eine gute glykämische Kontrolle zur Prävention und Progressionsvermeidung sowohl einer DSPN als auch einer CAN stellt somit eine der wesentlichen Empfehlung internationaler Leitlinien in der kausalen Therapie der diabetischen Polyneuropathie dar [7, 8].

Die entsprechende Rationale hierfür wurde vor allem mit dem Diabetes Control and Complications Trial (DCCT Trial) und der darauffolgenden Observationsstudie „Epidemiology of Diabetes Interventions and Complications“ (EDIC Trial) gelegt. Der DCCT Trial, als großangelegte prospektive Interventionsstudie, konnte eine 60 % Reduktion des Auftretens einer DSPN und eine 30 % Risikoreduktion einer CAN bei Personen mit Diabetes mellitus Typ 1 und optimierter glykämischer Kontrolle zeigen (HbA1c 7,4 % in der Gruppe mit intensivierter glykämischer Kontrolle vs. HbA1c 9,1 % in der konventionellen Gruppe) [5, 9]. Die darauffolgende Observationsstudie EDIC konnte auch nach einem follow Up von 13–14 Jahren eine geringere Rate an DSPN und CAN nach vormals intensivierter glykämischer Kontrolle nachweisen. Dabei zeigte sich ein anhaltend positiver Effekt unabhängig von der glykämischen Kontrolle zum Zeitpunkt des Follow-ups [4, 9].

Die Datenlage betreffend Diabetes mellitus Typ 2 ist hier weniger deutlich [1, 6]. Daten aus UKPDS, ACCORD und anderen Studien zum Outcome bei Diabetes mellitus Typ 2 zeigten zwar Hinweise, dass auch hier eine intensivierte glykämische Kontrolle mit einer Risikoreduktion für diabetische PNP vergesellschaftet ist, diese Ergebnisse zeigten sich allerdings zumeist nicht oder nur knapp signifikant [1, 6, 10]. Gründe hierfür könnten in unterschiedlichen Therapieregimen und womöglich auch in unterschiedlichen Subgruppen des Diabetes mellitus Typ 2 zu finden sein. So konnte die BARI 2D Study Group bereits 2009 zeigen, dass spezifische Therapien zur Erhöhung der Insulinsensitivität, wie Metformin oder Thiazolidindione, mit einer niedrigeren Inzidenz für eine sensorische diabetische Neuropathie einhergehen und umgekehrt scheint insbesondere in der erst kürzlich definierten Gruppe des schwer insulinresistenten Diabetes mellitus (SIRDD) ein höheres Risiko für die Entwicklung einer diabetischen Polyneuropathie zu bestehen [1, 11, 12].

### Medikamentöse Therapie der schmerzhaften diabetischen Polyneuropathie

#### Allgemeine Empfehlungen zur Therapie der schmerzhaften PNP

Grundsätzlich stehen systemisch wirksame und topische Therapeutika zur Verfügung, wobei auch eine gleichzeitige Anwendung beider Formen möglich ist [36].

Bei der Therapie der PNP muss dem Behandler bewusst sein, dass eine Schmerzfreiheit oft nicht erreicht werden kann. Dies soll auch dem Patienten so kommuniziert werden, um keine falschen Hoffnungen zu wecken. Bei allen medikamentösen Optionen spricht ein Teil der Patienten nur unzureichend auf die Therapie an oder leidet an nicht tolerierbaren Nebenwirkungen [37].

Vor Therapiebeginn sollte zur Verbesserung der Adhärenz über potenzielle Nebenwirkungen aufgeklärt werden. Des Weiteren sollte darüber aufgeklärt werden, dass die Wirkung erst nach Auftitration und Erreichen einer wirksamen Dosis und mit zeitlicher Verzögerung einsetzt, um das frühzeitige Absetzten von potenziell wirksamen Präparaten zu vermeiden. Die Wirksamkeit sollte unter ausreichender Dosierung erst nach zwei bis vier Wochen beurteilt werden. Es kann sinnvoll und effektiver sein, mehrere Medikamente zu kombinieren, da dadurch synergistisch schmerzhemmende Effekte auftreten können und die Einzeldosen niedriger bleiben können. Geduld und eine gute Kommunikation zwischen Behandelnden und Behandelten ist wichtig, um die individuell am besten geeignete Therapie zu finden. In die Diagnose und insbesondere auch die Wahl der Therapie sollten Patientinnen und Patienten partizipativ miteinbezogen werden [38].

Eine systemische Pharmakotherapie sollte immer wieder, spätestens alle drei bis sechs Monate, kritisch reflektiert werden [36].

Zusätzlich zu einer laufenden Pharmakotherapie ist ein multimodaler schmerztherapeutischer Ansatz häufig unverzichtbar, bei dem in spezialisierten Einrichtungen die medikamentösen Verfahren durch nichtmedikamentöse Verfahren aus der Physio‑, Sport- und Psychotherapie ergänzt werden [38].

Als realistische Therapieziele bei neuropathischen Schmerzen sind in der Regel anzustreben:

- Schmerzreduktion um ≥ 30 %
- Verbesserung der Schlafqualität
- Verbesserung der Lebensqualität
- Erhaltung der sozialen Aktivität und des sozialen Beziehungsgefüges
- Erhaltung der Arbeitsfähigkeit
- Verbesserung der Funktionalität

### Antikonvulsiva mit Wirkung auf neuronale Kalziumkanäle

#### Pregabalin/Gabapentin

Als systemische pharmakologische **Therapie erster Wahl** werden Antikonvulsiva mit Wirkung auf neuronale Kalziumkanäle empfohlen [37–39].

Das Antikonvulsivum Pregabalin hat eine schlaffördernde Wirkung und wirkt zusätzlich auch anxiolytisch. Pregabalin weist außerdem im Gegensatz zu Gabapentin eine höhere und dosisunabhängige biologische Verfügbarkeit und damit einen schnelleren Wirkeintritt bei ansonsten ähnlicher Wirksamkeit auf und wird daher im klinischen Alltag häufig bevorzugt [40].

In einer Metaanalyse fand sich ein besseres Ansprechen bei einer Tagesdosis von 600 mg im Vergleich zu der Tagesdosis von 300 mg [41]. Eine langsame Aufdosierung wird empfohlen [42].

In einer weiteren Studie bei 45 Patienten mit prädiabetischen Schmerzen wurde eine stärkere Schmerzreduktion unter Pregabalin erreicht, als mit Placebo [43].

Eine aktuelle Cochrane-Metaanalyse zur Wirkung von Gabapentin bei chronischen neuropathischen Schmerzen konnte eine signifikante Schmerzreduktion > 30 % nur in der Gruppe der Gabapentin behandelten Patienten bei schmerzhafter diabetischer Neuropathie festgestellt werden [44, 45]. Es gab mehr Nebenwirkungen, als im Placeboarm, jedoch kamen diese nur in 3 % vor.

**Pregabalin** wird angewendet zur Behandlung von peripheren und zentralen neuropathischen Schmerzen im Erwachsenenalter.

Die Dosis liegt zwischen 150 und 600 mg täglich, verabreicht in 2 oder 3 Einzeldosen [41, 46].

Bei eingeschränkter Nierenfunktion ist eine Dosisreduktion empfohlen.

**Gabapentin** ist zur Behandlung von peripheren neuropathischen Schmerzen wie schmerzhafter diabetischer Neuropathie und postherpetischer Neuralgie bei Erwachsenen indiziert.

Die Tagesdosis liegt zwischen 1200–3600 mg täglich, aufgeteilt in drei Gaben. Tagesdosis kann in 300 mg-Schritten alle 2–3 Tage bis zu einer maximalen Dosierung von 3600 mg/Tag erhöht werden [41, 46]. Bei eingeschränkter Nierenfunktion ist eine Dosisreduktion empfohlen.

##### Nebenwirkungen

Benommenheit, Schläfrigkeit, Schwindel, periphere Ödeme, Sehstörungen, Gewichtszunahme, Ataxie sowie Gangstörungen und vor allem bei älteren Patienten erhöhte Sturzneigung.

### Antikonvulsiva mit Wirkung auf Natriumkanäle

#### Carbamazepin

Wird als Substanz der **dritten Wahl** gezählt, da keine Bewertung der Evidenz aufgrund von einer unzureichenden Studienlage gemachte werden kann [47]. Positive Effekte zeigten Cross-over Studien, die jedoch schon über 40 Jahre alt sind [37, 48]. Auch die NeuPSIG-Empfehlungen [41] konnten aufgrund der mangelnden Datenqualität keine valide Bewertung für die Wirksamkeit von Carbamazepin bei der Behandlung neuropathischer Schmerzen erbringen.

#### Oxcarbazepin

Auch bei Oxacarbazepin ist die Studienlage zur Effektivität bei neuropathischen Schmerzen unzureichend. Es kann nicht generell zur Behandlung von neuropathischen Schmerzen empfohlen werden, kann jedoch im Einzelfall erwogen werden [37].

**Topiramat, Lamotrigin und Lacosamid **sollte nicht zur Therapie neuropathischer Schmerzen jeglicher Ursache eingesetzt werden [37, 49, 50]. In Metaanalysen konnte für Topiramat kein Wirknachweis bei diabetischer Polyneuropathie nachgewiesen werden [49].

### Antidepressiva

#### Trizyklische Antidepressiva (TCA)

Diese Substanzklasse wird als **Mittel der ersten Wahl** gesehen und soll zur Therapie von neuropathischen Schmerzen eingesetzt werden [38]. Trizyklische Antidepressiva haben vielfältige Wirkungsmechanismen. Sie haben keine direkten antinozizeptiven Eigenschaften und sind auch wirksam bei Patienten ohne Depressionen. Der Effekt auf die neuropathischen Schmerzen scheint früher und mit geringeren Dosierungen einzutreten als der Effekt auf die Depression [37, 51].

Trizyklische Antidepressiva sind dem Placebo überlegen [41, 52, 53]. Insgesamt werden die trizyklischen Antidepressiva als effektiv beurteilt, aber die Stärke der Evidenz wird als eher gering eingeschätzt [54].

Es sind sedierende (z. B. Amitriptylin) von nicht sedierenden (z. B. Clomipramin) TCA zu unterscheiden und entsprechend differenziert nach gewünschter Wirkung zu verordnen. Insbesondere bei Einschlafstörungen aufgrund neuropathischer Schmerzen kann Amitriptylin hilfreich sein.

Amitriptylin ist für die Therapie neuropathischer Schmerzen bei Erwachsenen zugelassen.

Die Startdosis liegt bei 10/12,5 mg oder 25 mg retardiert zur Nacht bei sedierenden TCA beziehungsweise morgens bei aktivierenden Wirkstoffen.

Steigerung: Dosissteigerung alle fünf bis sieben Tage um 10–25 mg (langsames Aufdosieren). Die empfohlene Höchstdosierung in der Schmerztherapie ist 75 mg, bei begleitenden Depressionen 150 mg am Tag. Je nach Wirkstoff erfolgt die Gabe retardiert einmalig oder verteilt auf zwei bis drei Tagesdosen.

##### Nebenwirkungen

Müdigkeit, Schwindel, Sedierung (Sturzgefahr), Miktions- und Akkomodationstörungen, Mundtrockenheit, Obstipation, Hypotonie, Gewichtszunahme, CYP-Interaktionen. Eine Kontraindikation besteht bei Reizleitungsstörungen, Herzinsuffizienz, Glaukom, Prostatahyperplasie und Thrombose. Regelmäßige EKG Kontrollen werden empfohlen [38, 46, 52].

#### Selektive Serotonin- und NoradrenalinWiederaufnahme-Hemmer (SSNRI)

SSNRI sind etwas besser verträglicher als TCA, aber möglicherweise etwas weniger effektiv [41].

#### Duloxetin

Diese Substanz wird als **Mittel der ersten Wahl** gesehen und soll zur Therapie von neuropathischen Schmerzen eingesetzt werden [37, 49, 52].

Die Analgesie wird durch die präsynaptische Wiederaufnahme-Hemmung der monoaminergen Neurotransmitter Serotonin und Noradrenalin und somit einer Verstärkung der deszendierenden schmerzhemmenden Bahnsysteme erklärt.

Es liegen mehrere Studien und Post-hoc-Analyse vor, die die Wirksamkeit von Duloxetin belegen [55–57]. Eine Dosierung unter 60 mg pro Tag brachte keinen wirksamen Effekt [58].

Duloxetin ist zur Behandlung von neuropathischem Schmerz, depressiven Erkrankungen und generalisierten Angststörungen zugelassen und sollte vor allem bei der diabetischen Polyneuropathie der Vorzug gegeben werden.

##### Startdosis

initial 30 mg.

Steigerung um 30 mg alle 4–7 Tage, Zieldosis: 60 mg/Tag morgens, MTD 120 mg.

##### Nebenwirkungen

Schwere Nebenwirkungen sind selten [58], Übelkeit, Kopfschmerzen, Schläfrigkeit, Mundtrockenheit, Hyperglykämien (bei Diabetes), CYP-Interaktionen, Dosisanpassung bei Rauchern (Wirkverlust) und bei Rauchstopp, keine Kombination mit Tramadol, Triptanen oder Johanniskrautpräparaten.

Einnahme mit Essen reduziert Übelkeit.

##### Gegenanzeigen

Die gleichzeitige Einnahme von Duloxetin mit nichtselektiven, irreversiblen Monoaminoxidase-Hemmern (MAO-Hemmern) ist kontraindiziert; schwere Nieren- und Leberfunktionsstörungen, Duloxetin darf nicht in Kombination mit Fluvoxamin, Ciprofloxacin oder Enoxacin (d. h. starken CYP1A2-Inhibitoren) angewendet werden.

#### Venlafaxin

Venlafaxin kann aufgrund der nicht ausreichenden Datenlage nicht zur Therapie von neuropathischen Schmerzen jeglicher Ursache empfohlen werden, kann aber in Einzelfällen als off-label Anwendung erwogen werden.

Noradrenerge und spezifisch serotonerge Antidepressiva (NaSSA) wie Mirtazapin wird laut deutscher Leitlinie nicht zum Einsatz bei neuropathischen Schmerzen jeglicher Ursache empfohlen. SSRI wie Citalopram/Escitalopram, Fluoxetin, Fluvoxamin oder Sertralin sollten nicht in der Therapie neuropathischer Schmerzen jeglicher Ursache eingesetzt werden [37]. Die Wirkung bei neuropathischem Schmerz konnte bisher nicht eindeutig nachgewiesen werden [39].

### Opioide

In der NeuPSIG-Leitlinie werden **niederpotente Opioide als zweite Wahl** und hochpotente Opioide als dritte Wahl empfohlen [41].

Bei unzureichender Schmerzlinderung unter Medikation erster Wahl, sollte leitliniengereicht zunächst mit einem niederpotenten Opioid (WHO Stufe-2-Analgetika) bzw., falls erforderlich, mit einem hochpotenten Opioid bzw. MOR/NRI (μ-Opoid Rezeptor Agonist und Noradrenalin Wiederaufnahme Hemmer Kombination zB Tapentadol) (WHO Stufe-3-Analgetikum) in retardierter Form begonnen werden. Opioide können auch verwendet werden, wenn eine Begleiterkrankung (z. B. Herzrhythmusstörungen), die gegen ein Erstlinientherapeutikum spricht, vorliegt. Zur Vermeidung einer opioidinduzierten Obstipation sollte immer auch an ein Laxans gedacht werden [59].

Die Expertinnen und Experten der interdisziplinären Gruppe der ÖSG sind der Ansicht, dass Opioidebesonders bei starken Schmerzen und für einen raschen therapeutischen Effekt ihren Stellenwert haben [38].

Opioide wirken als Agonisten an μ‑Opioidrezeptoren im zentralen Nervensystem. Einige Opioide wirken zusätzlich auf die endogene Schmerzmodulation. Je nach Wirksamkeit werden niederpotente und hochpotente Opioide unterschieden, wobei jeweils die Morphinäquivalenzdosis angegeben wird [49]. Eine antineuropathische Wirksamkeit weisen das schwache Opioid Tramadol sowie die starken Opioide Oxycodon, Buprenophin und Tapentadol auf [60].

#### Nebenwirkungen

Schwindel, Müdigkeit, Konzentrationsstörung, Obstipation, Toleranzentwicklung, opioidinduzierte Hyperalgesie, Suchtpotential.

#### Tramadol retard

Tramadol ist zugelassen zur Behandlung mäßig starker bis starker Schmerzen. Tramadol wirkt analgetisch und antitussiv, hat über einen großen Dosisbereich keine atemdepressive Wirkung und beeinflusst auch nicht die gastrointestinale Motilität [61].

Die Evidenz ist aufgrund kleiner Teilnehmerzahlen pro Studie und dem resultierenden Risiko von Verzerrungen insgesamt gering, so dass der positive Effekt von Tramadol möglicherweise überschätzt wird [62].

##### Startdosis

2 (–3) × 50–100 mg oder 1 × 150 mg.

##### Zieldosis

100–200 mg/d, in 2 (–3) Einzeldosen, MTD 600 mg/d.

#### Tapentadol retard

Tapentadol wird angewendet zur Behandlung erwachsener Patienten mit mäßig starken bis starken akuten oder chronischen Schmerzen, die nur mit Opioidanalgetika angemessen behandelt werden können.

In einer Analyse zweier placebokontrollierter Studien zur Behandlung der diabetischen Polyneuropathie konnte durch den Einsatz von Tapentadol eine signifikante Schmerzlinderung erzielt werden [49]. Insgesamt ist jedoch ist die Datenlage zur Therapie des neuropathischen Schmerzes nicht ausreichend [63].

##### Startdosis

2 (–3) × 50 mg.

##### Zieldosis

100–200 mg/d in 2 (–3) Einzeldosen, MTD: 500 mg/d.

#### Oxycodon retard

Oxycodon wirkt aktivierend an µ‑, κ‑ und δ‑Opioidrezeptoren in Gehirn und Rückenmark. Seine therapeutische Wirkung ist vorwiegend analgetisch und sedierend. Der Wirkstoff gehört zu den Dihydroderivaten des Morphins. Es besitzt eine 3 mal höhere orale Bioverfügbarkeit als andere Opioide [61]. Aus den vorliegenden Daten lässt sich ein moderater Therapievorteil ableiten.

##### Startdosis

2 (–3) × 5–10 mg.

##### Zieldosis

individuell, MTD bei Tumorpatienten 400 mg/d.

#### Hydromorphon

Durch die Bindung an µ‑Opioidrezeptoren verringert es einerseits die Weiterleitung von Schmerzsignalen in den Nervenzellen und setzt andererseits die Schmerzwahrnehmung im Gehirn (Thalamus und limbisches System) herab. Trotz guter methodischer Qualität kann aufgrund der vorliegenden Daten nicht beurteilt werden, ob Hydromorphon einen Effekt in der Behandlung neuropathischer Schmerzen hat [37].

### Cannabinoide

Können nicht generell zur Therapie neuropathischer Schmerzen empfohlen werden, da ihr Effekt eher gering ausgeprägt und die Nebenwirkungsrate hoch ist [37, 49]. Kontroverse Ergebnisse, kleine Studie und kurze Studiendauer lassen keine eindeutige Aussage zu. Sie kommen als Drittlinien- bzw. Add-on-Therapie nach Ausschöpfung der anderen empfohlenen Maßnahmen in Betracht [64].

### Alpha-Liponsäure

Alpha-Liponsäure kann nicht zur Therapie neuropathischer Schmerzen jeglicher Ursache empfohlen werden. Ein Effekt bei der diabetischen Neuropathie kann nicht ausgeschlossen werden. Die Evidenzlage ist allerdings nicht ausreichend, um den Einsatz bei der diabetischen Neuropathie generell zu empfehlen [37].

Die Anwendung der i.v. Verabreichung konnte bessere Ergebnisse bringen als die orale [65]. Sie wird vor allem bei der Indikation der diabetischen Polyneuropathie angewandt.

#### Dosierung

TD 600 mg über 3 Wochen.

#### Nebenwirkungen

Sehr selten Übelkeit, Erbrechen, Magen/Darm, ZNS (Schwindel), Vegetativum, Allergie/Anaphylaxie.

### Nicht Opioidanalgetika

NSAR, Cox-2-Inhibitoren, Paracetamol, Metamizol etc. sollten nicht zur Therapie chronisch neuropathischer Schmerzen eingesetzt werden. Sie können bei Langzeitanwendung gefährliche Nebenwirkungen aufweisen. Die Datenlage zu diesen Medikamenten ist in dieser Indikation gering und brachte keine signifikante Schmerzreduktion [66].

### Topische Therapie

Bei lokalisierten neuropathischen Schmerzen sollte auch eine topische Therapie bereits frühzeitig in Betracht gezogen werden, um insbesondere zentralnervöse Nebenwirkungen und Arzneimittelinteraktionen möglichst zu vermeiden und als adjuvante Therapie die Dosis einer systemischen Medikation zu reduzieren.

#### Lidocain-Pflaster

Lidocain unterbindet über eine Blockade der spannungsabhängigen Natriumkanäle die Entstehung ektoper Aktionspotenziale [67, 68]. Das Lidocainpflaster ist zur Linderung der Symptome neuropathischer Schmerzen nach einer Herpes-Zoster-Infektion bei Erwachsenen zur Mono- oder Kombinationstherapie zugelassen [41]. Die aktuelle DGN-Leitlinie vom Mai 2019 empfiehlt es grundsätzlich als Zweitlinientherapie bei lokalisierten neuropathischen Schmerzen.

##### Startdosis

5 % (700 mg) Pflaster; 10 × 14 cm; 1 × tgl.

##### Zieldosis

1–3 Pflaster tgl.

##### Nebenwirkungen

Erythem und Unverträglichkeiten am Applikationsort, kaum systemische Nebenwirkungen oder Medikamenteninteraktionen.

#### Capsaicinpflaster

Die S2-Leitlinie empfiehlt das Hochdosispflaster als zweite Wahl zur Therapie neuropathischer Schmerzen, bei lokalisierten Schmerzen auch als Primärtherapie [37]. Allgemein werden Capsaicinpflaster hinsichtlich ihres schmerzlindernden Effekts in verschiedenen Übersichtsarbeiten als vergleichbar zu anderen Therapieansätzen bewertet [69].

Die Indikation umfasst alle peripheren neuropathischen Schmerzätiologien bei Erwachsenen [70].

##### Dosierung

179 mg (8 %) kutanes Pflaster 14 × 20 cm; Einmalige Anwendung, evtl. Wiederholung nach 90 Tagen möglich; Anwendung nur unter Aufsicht medizinischen Personals. Entfernung des Pflasters nach 30 min.

1–4 Pflaster pro Anwendung. Eine erneute Anwendung nach weniger als 3 Monaten kann für einzelne Patienten in Betracht gezogen werden (Mindestintervall: 60 Tage).

##### Nebenwirkungen

lokale Hautreaktion (Jucken, Brennen, Pusteln), vermindertes Geschmacksempfinden, Rötung, Tachycardie/Bradycardie, Blutdruckanstieg.

#### Transkutane Elektrische Nervenstimulation (TENS)

Kann aufgrund der fehlenden Evidenz nicht empfohlen werden; da Einzelstudien eine Wirksamkeit nahelegen, kann der Einsatz in Einzelfällen erwogen werden [71].

### Multimodale Schmerztherapie

#### Psychotherapeutische Interventionen

Psychotherapeutische Behandlungsansätze können in der Therapie neuropathischer Schmerzen jeglicher Ursache eingesetzt werden. Obwohl die Evidenz der Schmerzreduktion nicht belegt ist, stellt die Schmerzpsychotherapie eine wichtige Therapieoption dar [37].

#### Physiotherapie

Sollte zur Ergänzung einer Medikation zur Verbesserung von Stand, Gang und Gleichgewicht und zum gezielten Training der Muskelkraft erfolgen [52, 72]. Die Evidenzlage der Physiotherapie ist bisher gering, es gibt jedoch zahlreiche Studien zur Wirksamkeit der einzelnen Techniken.

#### Ergotherapie

Zur Verbesserung der Feinmotorik können ergotherapeutische Maßnahmen eingesetzt werden.

#### Akupunktur

Dass Akupunktur Nerven schützen kann, zeigt eine aktuelle Studie bei chemotherapie-induzierter Polyneuropathie. Wie die gezielten Reize der Akupunktur wirken, ist noch unklar. Mit einer Wärmeleitkamera lässt sich zeigen, dass die behandelten Regionen während einer Akupunktur durchblutet sind. Aufgrund der geringen Teilnehmerzahl, dem kurzen Follow-up und dem monozentrischen Design müssen weitere Studien die Daten belegen [73].

Eine Meta-Analyse von 25 randomisierten Studien in China kam zum Schluss, dass Akupunktur die globalen Schmerzsymptome verbesserte, im Gegensatz zu Vitamin B-Gabe oder im Vergleich zu keiner Therapie [74].

### Invasive Verfahren

#### Spinal Cord Stimulation (SCS)

SCS kann in Erwägung gezogen werden, wenn andere Therapieversuche keine ausreichende Schmerzerleichterung bringen [75, 76]. Diese Methode ist für den Einsatz bei therapierefraktärem Schmerz bei Polyneuropathie empfohlen [77].

#### Spinalganglienstimulation (DRG)

Diese kann bei therapierefraktärem Schmerz in Erwägung gezogen werden, jedoch findet man in der Literatur nur limitierte Daten mit einem anzunehmenden Bias. Neue, unabhängige Daten sind wünschenswert [78, 79].

#### Zusammenfassung der medikamentösen Therapie der schmerzhaften Polyneuropathie (Tab. 1)

**Medikamente und Dosierungen bei Neuropathischen Schmerzen **[36, 38].

Bitte jeweils die Fachinformationen beachten!

**Table 1:** 

ARZNEISTOFF	STARTDOSIS	AUFDOSIERUNG ZIELDOSIS (ZD) MAXIMALDOSIS (MTD)	BESONDERHEITEN UND WICHTIGE NEBENWIRKUNGEN
*Medikation 1. WAHL:*			
Gabapentin	3 × 100mg bzw. 1 × 300mg (Beginn mit abendlicher Dosis)	Täglich um 300 mg steigern bis 1200 mg/d, dann falls erforderlich wöchentlich um 600 mg steigern ZD 1200–3600 mg/d, 3–4 Dosen MTD 3600 mg/d	Müdigkeit, Schwindel, Gangunsicherheit, periphere Ödeme, kaum Interaktionen, Dosis an Nierenfunktion anpassen, verzögerter Wirkbeginn
Pregabalin	2 × 50–75 mg (Beginn mit abendl. Dosis)	Nach 3–7 Tagen Steigerung um 50–75 mg auf 150 mg/d, dann falls erforderlich wöchentlich um 150 mg steigern ZD 150–600 mg/d, 2 Dosen MTD 600 mg/d	Müdigkeit, Schwindel, Gangunsicherheit, periphere Ödeme, Gewichtszunahme, wirkt anxiolytisch, kaum Interaktionen, Dosis an Nierenfunktion anpassen, verzögerter Wirkbeginn
Amitriptylin	10–25 mg (abends)	10–25 mg alle 7 Tage ZD 25–75 mg/d, bei Depression 75–150 mg/d, Einzeldosis MTD 150 mg/d	Müdigkeit, Schwindel, Sedierung, Sturzgefahr, Miktions- und Akkomodationsstörungen, Hypotonie, Gewichtszunahme, CYP-Interaktionen, langsame Aufdosierung, kardiale NW (EKG Kontrolle)
Duloxetin	30–60 mg (morgens)	30 mg alle 4–7 Tage ZD 60 mg/d morgens MTD 120 mg/d	Übelkeit und Erbrechen, Mundtrockenheit, Blutdruckanstieg, CYP-Interaktionen, Dosisanpassung bei Rauchern, keine Kombi mit Tramadol, Triptanen oder Johanniskraut
*Medikation 2. WAHL*			
Lidocain Pflaster	5 % (700 mg) 1 × tgl. Bis zu 12 h Pause	1–3 Pflaster täglich	Erythem und Unverträglichkeitsreaktionen am Applikationsort, kaum systemische NW
Capsaicin Pflaster	8 % (179 mg) 1 × 30 min; 90 Tage Pause	1–4 Pflaster pro Anwendung alle 3 Monate oder später	Erythem, Rötung, Brennschmerz und Unverträglichkeitsreaktionen am Applikationsort, temporäre Schmerzzunahme ggf. Blutdruckanstieg, keine systemischen Nebenwirkungen oder Medikamentenwechselwirkungen
Tramadol retard	2–3 × 50–100 mg	50–100 mg alle 3–4 Tage ZD 100–200 mg/d, 2–3 Dosen MTD 600 mg/d	Übelkeit, Hypotonie, Dosisreduktion bei eingeschränkter Nierenfunktion, keine Kombi mit serotonergen Substanzen oder Duloxetin
Tapentadol retard	2 × 50 mg	100 mg alle 3–4 Tage ZD 100–200 mg/d, 2–3 Dosen MTD 500 mg/d	Übliche Opioid-NW bei geringerer Obstipation und Absetzproblematik
*Medikation 3. WAHL*			
Carbamazepin	100–400 mg (abends)	200 mg alle 3–7 Tage ZD 600 mg/d, 2 Dosen MTD 1200 mg/d	Kognitive Beeinträchtigung, Blutbildveränderungen, Leberschäden, Hyponatriämie, Hautausschlag, Medikamenteninteraktionen wegen Enzyminduktion, langsame Aufdosierung nötig
Oxycodon retard	2–3 × 5–10 mg	Individuell MTD Tumorpat. 400 mg/d	Übliche Opioid-NW, Dosisreduktion bei Leber- und Niereninsuffizienz
Buprenorphin TTS	5–20 µg/h	Individuell	Übliche Opioid-NW, keine Dosisreduktion bei eingeschränkter Nierenfunktion
Botolinumtoxin	–	–	–

### Therapie der autonomen Neuropathie

Auch bei der autonomen Neuropathie gelten die gleichen Grundsätze, wie bei der diabetischen sensomotorischen Neuropathie. Es liegen leider nur wenige größere kontrollierte Studien zur Pharmakotherapie der symptomatischen autonomen Neuropathie, mit Ausnahme der erektilen Dysfunktion, vor.

Die symptomatische Pharmakotherapie an verschiedenen Organ- und Funktionssystemen ist in der Regel durch entsprechende Spezialisten im Rahmen der interdisziplinären Zusammenarbeit einzuleiten [47].

#### Kardiovaskuläre autonome Neuropathie (CAN)

Betablocker mit intrinsischer sympathomimetischer Aktivität (z. B. Pindolol) und trizyklische Antidepressiva in antidepressiv wirksamer Dosierung (z. B. Amitriptylin, Imipramin) sollten bei Patienten mit KADN aufgrund ihres ungünstigen Einflusses auf die HRV und der erhöhten Gefahr von Herzrhythmusstörungen nicht gegeben werden. Körperliches Training, Kompressionsstrümpfe oder liberalisierter Kochsalzkonsum werden bei orthostatischer Hypotonie empfohlen. Bei Sinustachycardie sollten kardioselektive Betablocker verschrieben werden [47, 80].

#### Autonome Neuropathie am Gastrointestinaltrakt

Messbare gastrointestinale Funktionsstörungen, die jedoch zu keinerlei Beschwerden oder Beeinträchtigungen führen, sind nicht therapiebedürftig. Bei diabetischer Gastropathie in Form einer beschleunigten Magenentleerung sollten mehrere kleine Mahlzeiten mit Vermeidung von rasch resorbierbaren Kohlenhydraten empfohlen werden. Patienten mit einer diabetischen Gastroparese soll eine Umstellung der Ernährung angeraten werden, d. h. kleine, über den Tag verteilte Mahlzeiten mit reduzierter Fettzufuhr und wenig Ballaststoffen. Prokinetika (Metoclopramid und Domperidon) können als Off-Label-Use probiert werden. Teilweise werden auch Antiemetika (Antihistaminika, 5‑HT3-Antagonisten) bei ausgeprägter Übelkeit und Erbrechen verordnet.

Bei Reflux wird die Verwendung von Protonenpumpenhemmern empfohlen [80].

Allgemeine Maßnahmen wie ausreichendes Kauen und aufrechte Körperhaltung beim Essen sind die Basis der Therapie.

#### Autonome Neuropathie am Urogenitaltrakt

Diabetische Zystopathie kann durch Verhaltensmodifikation, Elektrostimulation oder Biofeedback oft positiv beeinflusst werden. Insgesamt sind die Symptome und die Folgen der diabetischen Zystopathie durch eine medikamentöse Therapie nur eingeschränkt beeinflussbar. Bei einer instabilen Stoffwechselsituation und bei manifesten Zystopathie sind Harnwegsinfektionen bei Menschen mit Diabetes mellitus als kompliziert zu werten [18].

Bei erektiler Dysfunktion ist eine Vermeidung medikamentöser Nebenwirkungen oberstes Ziel. Als Therapie der ersten Wahl werden 5‑Phosphodiesterase-Hemmer (Sildenafil, Tadalafil, Vardenafil, Avanafil) empfohlen. Bei unzureichender Wirkung stehen Erektionshilfen oder Schwellkörperinjektionstherapien zur Verfügung.

## Diabetischer Fuß

Das diabetische Fußsyndrom gehört zu den schwerwiegendsten Folgeerkrankungen des Diabetes mellitus. Definiert ist das diabetische Fußsyndrom als Ulzeration, Infektion oder Gewebsdestruktion an der unteren Extremität bei Menschen mit Diabetes mellitus verursacht durch eine Neuropathie und/oder peripher arterielle Verschlusskrankheit [1].

19–34 % aller Menschen mit Diabetes entwickeln im Laufe der Erkrankung ein Ulkus und die weltweite Prävalenz liegt bei 2–10 % [81].

Das diabetische Fußsyndrom ist die Hauptursache für nichttraumatische Amputationen, rund 40–60 % aller nicht traumatischen Amputationen der unteren Extremität werden bei Menschen mit Diabetes durchgeführt [82].

### Pathogenese

Die Pathogenese des diabetischen Fußsyndroms ist multifaktoriell. 90 % aller Menschen mit Diabetes mellitus und diabetischem Fußsyndrom haben eine sensomotorische Neuropathie. Eine peripher arterielle Verschluss Krankheit liegt bei mindestens 50 % der Fälle vor [83].

Der Verlust der Warn/Schutzfunktion durch die eingeschränkte oder fehlende Sensibilität (LOPS = Loss of protective sensation), Fußdeformitäten und eine eingeschränkte Mobilität der Gelenke führt zu einer abnormalen biomechanischen Belastung des Fußes. Dieser erhöhte Druck führt zur Bildung von Hyperkeratosen (Kallus) mit möglicher Blasenbildung oder Einblutung und daraus resultierenden Ulzerationen.

Neben der abnormen biomechanischen Belastung mit konsekutiven Ulzerationen sind Mikrotraumata eine weitere häufige Ursache von Ulzerationen. Mikrotraumata entstehen meist durch nicht passendes Schuhwerk, eine falsche Fußpflege (Verletzung beim Kürzen der Nägel) oder thermische Verletzungen durch Verbrennungen (z. B. durch zu heißes Badewasser oder Sauna) aber auch Erfrierungen.

Die Neuropathie spielt aufgrund ihrer zusätzlich bestehenden psychologischen Komponente eine wesentliche Rolle.

Die peripher arterielle Verschlusskrankheit (PAVK) ist ein wichtiger Risikofaktor für eine schlechte Wundheilung und ein hohes Amputationsrisiko. Bei Patienten mit neuro-ischämischen Ulzerationen ist zu beachten, dass meist die klassische Symptomatik der Claudicato intermittens aufgrund der bestehenden Neuropathie fehlt. Daher muss jede Ulzeration beim Menschen mit Diabetes angiologisch abgeklärt werden.

Ein pathologischer Arm Bein Index ABI ist prädiktiv für kardiovaskuläre Ereignisse und geht mit einer erhöhten kardiovaskulären Mortalität einher [84]. Bei nicht-konklusivem ABI (Tab. 2) sollte als nächster diagnostischer Schritt eine Duplexsonographie der peripheren Arterien inklusive Analyse des Dopplerspektrums erfolgen [85]. Sollte auch die Duplexsonographie zu keiner definitiven Diagnose führen bzw. eine Revaskularisierung angestrebt werden, ist die Durchführung nicht-invasiver und invasiver angiographischer Verfahren (MR-Angiographie, CT-Angiographie, konventionelle intraarterielle Angiographie) indiziert. Das für den individuellen Patienten optimale bildgebende Verfahren wird in Abhängigkeit von Co-Morbiditäten (z. B. Niereninsuffizienz) und lokaler Expertise gewählt [84].

**Table 2:** 

Normal	0,91–1,30
Leichte Obstruktion	0,70–0,90
Moderate Obstruktion	0,40–0,69
Schwere Obstruktion	< 0,40
Schlechte Kompressionsfähigkeit	> 1,30

### Prävention

Primäres Ziel bei der Behandlung von Menschen mit Diabetes ist die Prävention von mikro und makrovaskulären Spätkomplikationen durch eine optimale Stoffwechselkontrolle und Optimierung der kardiovaskulären Risikofaktoren.

Zur Prävention des diabetischen Fußsyndroms gibt es 5 Schlüsselelemente:

1. Identifikation eines Risikofußes
2. Regelmäßige Inspektion und Untersuchung des Risikofußes
3. Schulung des Patienten, dessen Angehörige und im Gesundheitsbereich arbeitende Personen
4. Tragen von geeignetem Schuhwerk
5. Behandlung von präulzerösen Läsionen wie z. B.: Hornhautschwielen

Die Identifikation eines Risikofußes erfolgt nach Tab. 3. Eine fehlende protektive Wahrnehmung (LOPS = Loss of protective sensation) wird im Regelfall durch die Anamnese und durch ein Neuropathie Screening diagnostiziert. Meist erfolgt in der Praxis aus Zeitgründen das Neuropathie Screening durch Bestimmung des Vibrationsempfinden und des Berührungsempfinden mittels Monofilament. Andere Untersuchungen hinsichtlich der Neuropathie sind nur in Ausnahmefällen notwendig. Liegt eine bekannte Neuropathie, PAVK, Fußdeformität, stattgehabtes Ulkus/Amputation vor, besteht immer ein erhöhtes Ulkusrisiko. Ein Neuropathiescreening muss dann nicht mehr durchgeführt werden. Die Füße müssen jedoch regelmäßig von medizinischem Fachpersonal inspiziert werden. Hinsichtlich einer möglichen PAD sollten bei vermeintlich gesundem Fuß zumindest einmal jährlich eine Überprüfung der Fußpulse stattfinden.

**Table 3:** 

Kategorie	Ulkusrisiko	Charakteristik	Screening^a^
0	Sehr niedrig	Keine fehlende protektive Wahrnehmung	Einmal jährlich
1	Niedrig	Fehlende protektive Wahrnehmung *oder* Periphere Durchblutungsstörung	Alle 6–12 Monate
2	Moderat	Fehlende protektive Wahrnehmung und periphere Durchblutungsstörung *oder* Fehlende protektive Wahrnehmung und Fußdeformation *oder* Periphere Durchblutungsstörung und Fußdeformation	Alle 3–6 Monate
3	Hoch	Fehlende protektive Wahrnehmung oder periphere Durchblutungsstörung und ein oder mehrere folgende Faktoren:	Alle 1–3 Monate
		Früherer Fußulkus	
		Amputation unterer Extremität (klein oder groß)	
		Terminale Niereninsuffizienz	

Die Screening Intervalle basieren auf Expertenmeinung.

Aufgrund des Neglects für ihre Erkrankung sollten bei der Schulung beziehungsweise Aufklärung über das diabetische Fußsyndrom, wenn möglich Angehörige einbezogen werden. Präventiv sollten die Füße einmal täglich vom Betroffenen oder mit Hilfe von Angehörigen kontrolliert werden (auch die Zehenzwischenräume). Die Hautpflege erfolgt mit ureahaltigen Pflegecremen. Geeignetes Schuhwerk ist zur Prävention essentiell. Prinzipiell neigen Menschen mit Neuropathie dazu zu enges Schuhwerk zu bevorzugen, da sie das Gefühl haben damit besser gehen zu können. Ist die Fußdeformität nicht zu ausgeprägt, sind Konfektionsschuhe mit Weichbettungseinlagen geeignet. Bei einer ausgeprägten Fußdeformität müssen orthopädische Maßschuhe angepasst werden. Vor dem Anziehen der Schuhe ist der Schuh auf Fremdkörper zu überprüfen. Zu vermeiden sind barfuß oder mit dünnen Socken zu gehen, da Fremdkörper zu Verletzungen führen können. Wärmedecken und ein zu heißes Fußbad gehen mit einer hohen Verbrennungsgefahr einher. Auf eine korrekte und verletzungsfreie Nagelpflege ist zu achten [1, 83].

Als präulzeröse Läsionen bezeichnet man Hyperkeratosen oder Nagelfehlstellungen. Insbesondere Hyperkeratosen müssen regelmäßig entfernt werden. Dies sollte durch eine professionelle Fußpflege erfolgen. Aufgrund des häufig vorhandenen sozioökonomischen Nachteils von Menschen mit Diabetes ist dies nicht immer möglich.

Bei der klinischen Untersuchung von Füßen in der Risikokategorie 1–3 sollte auf folgendes geachtet werden:

1. Hautfarbe, Temperatur, Hornhautschwielen, Ödeme, Muskelatrophien
2. Fußdeformitäten wie Krallenzehen, verändertes Längs- und Quegewölbe, prominenter Mittelfußbereich, abnorme Knochenvorsrpünge
3. Ist korrektes Schuhwerk vorhanden
4. Adäquate Fußhygiene
5. Evaluierung ob eine korrekte Fußpflege durch den Patienten selbst durchgeführt werden kann
6. Aktuelles Wissen zum diabetischen Fußsyndrom

## Evaluierung von Fußulzerationen

Das diabetische Fußsyndrom sollte standardisiert evaluiert werden, um eine optimale Therapie zu ermöglichen.

### Klassifikation von Ulzerationen

Es gibt verschieden Scores um Ulzerationen bei Menschen mit Diabetes zu klassifizieren. Von der IWGDF (International Working Group on the Diabetic Foot) [86] wird der SINBAD score empfohlen, da er einfach anzuwenden ist (Tab. 4).

**Table 4:** 

Kategorie	Definition	Punkte
Ulkuslokalisation	Vorfuß	0
	Mittelfuß oder Rückfuß	1
Ischämie	Zumindest ein Puls tastbar	0
	Klinischer Hinweis auf Durchblutungsstörung	1
Neuropathie	Keine Neuropathie	0
	Neuropathie	1
Infektion	Keine Infektion	0
	Infektion	1
Ulklusgröße	Ulkus keiner 1 cm^2^	0
	Ulkus größer 1 cm^2^	1
Ulkustiefe	Oberflächliches Ulkus	0
	Ulkus mit Sehen oder Muskelbeteiligung oder tiefer	1
Maximale Punktezahl		6

In einer multizentrischen Studie zeigte sich eine deutliche Verlängerung in der Zeit bis zur Abheilung bei einem SINBAD Score von 2–3. Bei einem Score von 3 und mehr ist eine Abheilung oft unwahrscheinlich [87].

Durch die klinische Untersuchung kann zumindest teilweise ein Ulkus einer im Vordergrund stehenden Pathophysiologie zugeordnet werden Ein neuropathisches Ulkus präsentiert sich an bekannten Prädilektionsstellen vor allem im Bereich der Köpfe der Os metatarsalia oder an den Zehenspitzen beziehungsweise dorsal an den Interphalangeal Gelenken. Die Ulzerationen sind meist rund, wie ausgestanzt mit einer umgebenden Hyperkeratose. Die Hyperkeratose ist Ausdruck einer Druckbelastung. Das ischämische Ulkus ist meist akral lokalisiert im Sinne einer Nekrose. Da neuro-ischämische Ulzerationen häufig sind und es klinisch kaum möglich ist, dies zu differenzieren, muss wie schon beschrieben jedes Ulkus angiologisch abgeklärt werden.

### Infektion

**Figure 1:**
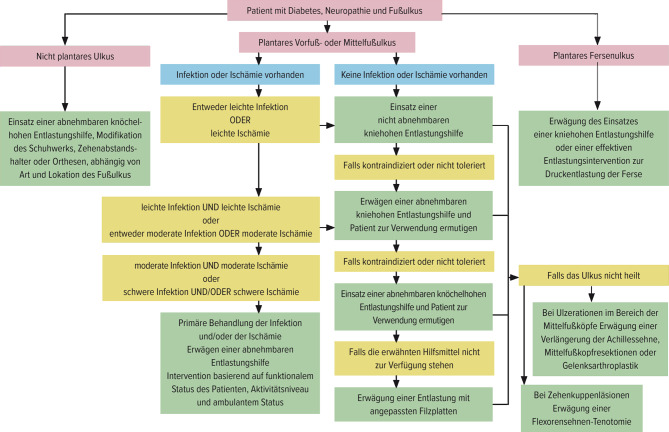

Jede Infektion kann den Fuß des Menschen mit Diabetes gefährden. Daher müssen Ulzerationen regelmäßig klinisch auf das Vorhandensein einer Infektion evaluiert werden. Da alle Ulzerationen kolonisiert sind, basiert die Diagnose einer Infektion auf dem Vorhandensein von mindesten zwei entsprechenden klinischen Zeichen (Rötung, Schwellung, Überwärmung, Schmerz). Sind Infektionszeichen vorhanden erfolgt die Einteilung der Infektion nach Abb. 1. Infektionen müssen prompt therapiert werden.

### Osteomyelitis (OM)

Infektionen des Knochens per continuitatem am Fuß treten häufig auf. Die knöchernen Strukturen liegen nahe an der Oberfläche. Die Diagnose gestaltet sich immer wieder schwierig. Beim DFS ist eine „Probe to bone“ obligatorisch. Mit einer Sonde oder ähnlichem Instrument wird versucht, ob der Knochen tastbar ist. Ist kein Knochen tastbar, ist eine Osteomyelitis eher unwahrscheinlich. Die „Probe to bone“ ist von der Erfahrung des Untersuchers abhängig und daher schwer reproduzierbar.

Besteht der Verdacht auf eine OM ist der nächste diagnostische Schritt ein konventionelles Röntgen, da überall verfügbar. Kann im Röntgen die Diagnose nicht bestätigt werden, aber der Verdacht auf eine Knochenbeteiligung weiterhin besteht, ist die Magnetresonanzuntersuchung das Mittel der Wahl mit der höchsten Spezifität und Sensitivität [88, 89].

## Therapie

Aufgrund seiner Komplexität benötigt das diabetische Fußsyndrom immer einen multiprofessionellen Behandlungsansatz.

### Therapieziel

Die Definition eines auf das Individuum zugeschnittenen Therapieziels abhängig von Begleiterkrankungen und der Lebenserwartung ist essentiell. Neben der Abheilung einer Ulzeration kann auch die Erhaltung der Mobilität beziehungsweise eine Amputations- und Infektionsvermeidung im Vordergrund stehen.

### Druckentlastung

**Figure 2:**
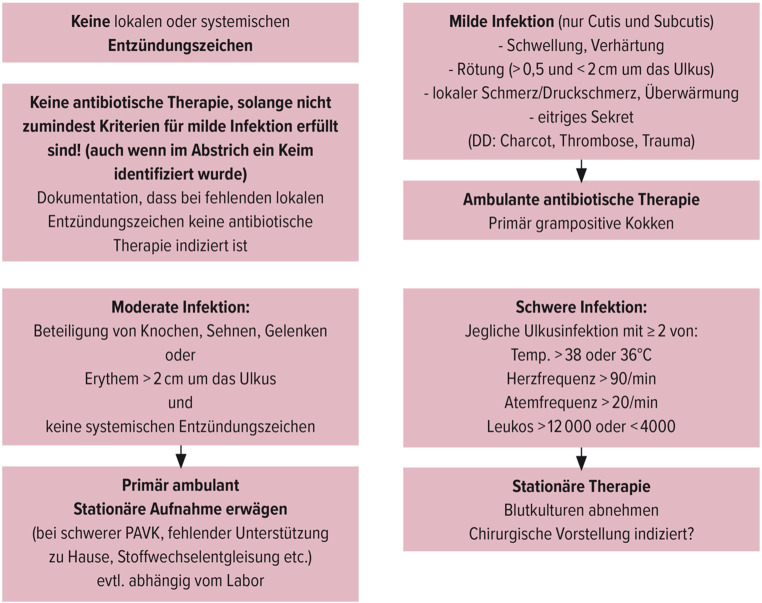

Der Grundpfeiler in der Behandlung von Ulzerationen, welche durch vermehrten biomechanischen Stress verursacht wurden, ist die Druckentlastung der Läsion. Ist das Therapieziel die Abheilung der Läsion ist dies die wichtigste und zugleich im täglichen Leben am schwersten umsetzbare therapeutische Maßnahme Es gilt der Leitsatz: „Hit hard and early“ Der Goldstandard bei der Entlastung ist ein Vollkontaktgips. Seine Überlegenheit wurde in mehreren randomisiert kontrollierten Studien nachgewiesen. Dieser garantiert eine Druckentlastung 24 h am Tag. Wird dieser vom Patienten mit einem DFS nicht akzeptiert oder toleriert kann eine abnehmbare Vakuum-Schiene (z. B VACO®Cast) in Betracht gezogen werden. In der Step down Therapie folgen als Empfehlung anpassbare Verbandsschuhe (z. B.: WCS® Verband Schuh). Eine individualisierte Druckentlastung kann nach Abb. 2 erfolgen. Jegliche Form von Vorfuß-Entlastungsschuhen sind aufgrund der Sturzgefahr obsolet.

### Peripher arterielle Verschlußkrankheit

Nach weiterführender stadiengerechter Diagnostik und konklusiver Darstellung der arteriellen Strombahn (Duplexsonographie, MRA, CTA) gilt die Revaskularisation als zentrales Ziel bei vorhandenen Stenosen. Der optimale Behandlungsmodus (endovaskulär vs. operativ vs. Hybrid-Verfahren) sollte in einem interdisziplinären Gefäßboard festgelegt werden.

### Infektion

Milde und moderate Infektionen werden im Regelfall 2 Wochen und eine schwere Infektion 3 Wochen antibiotisch je nach vorhandener Resistenzlage therapiert. Wenn möglich sollte immer ein Keimnachweis angestrebt werden. Eine Gewebsprobe ist einem tiefen Wundabstrich vorzuziehen. Bei einer milden Infektion sollte eine empirische Antibiose mit Substanzen gegen Staphylokokkus aureus und Streptokokken begonnen werden. Bei moderaten bis schweren Infektionen muss sowohl das gram-positive, als auch das gram-negative Spektrum an möglichen Keimen abgedeckt werden. Gegebenenfalls muss auch eine chirurgische Nekrektomie evaluiert werden [90].

Die Therapiedauer einer Knocheninfektion liegt in der Regel bei 6 Wochen. Auch hier sollte eine Keimgewinnung mit Antibiogramm angestrebt werden. Führt eine antibiotische Therapie nicht zum Erfolg, muss eine chirurgische Sanierung der Osteomyelitis je nach Therapieziel des Patienten in Betracht gezogen werden. Bei Amputation muss immer bedacht werden, dass selbst Minor Amputationen wieder zu neuen Ulzerationen durch eine veränderte Biomechanik am Fuß führen können. Man spricht hierbei auch von Transfer-Ulzerationen.

### Lokaltherapie

Die feuchte Wundbehandlung gehört zu den Standards bei der Behandlung von Ulzerationen. Die Evidenz zu Vorteilen einzelner Verbandsstoffe ist sehr dünn. Primär gilt, dass die Verbandsauswahl je nach Wundstadium und Exsudation erfolgt. Trockene Nekrosen sollten trocken gehalten werden.

Vorhandene Hyperkeratosen, Beläge und nekrotisches Gewebe sollten mechanisch entfernt werden. Beim scharfen Debridement zeigte eine Studie einen Vorteil gegenüber der einer Standardtherapie. Ein enzymatisches Debridement brachte im Vergleich zu Hydrogel keinen Vorteil. Studien zur Madentherapie sind mit einem hohen Bias verbunden und somit ist keine Beurteilung hinsichtlich eines Zusatznutzen möglich [91]. Auch Wundauflagen mit Wirkstoffen inklusive Silber zeigten keinen eindeutigen vorteilhaften Nutzen. Ein Cochrane Review aus dem Jahre 2017 bestätigt lediglich, dass antimikrobielle Wundauflagen die Heilungsrate verbessern könnten. Der Evidenzgrad ist jedoch niedrig [92].

Eine Wundauflage mit einer Sucrose Octasulfat Imprägnierung zeigte bei nicht infizierten neuroischämischen Fußulzerationen einen signifikanten Vorteil [93]. Biotechnologische Ansätze wie Hautersatz oder allogenes plättchenreiches Plasma zeigten Vorteile bei geringer Qualität der Evidenz[94].

Die Stosswellentherapie zeigte in einer Metaanalyse einen leichten Vorteil gegenüber der Standardtherapie [95, 96].

Die hyperbare Sauerstofftherapie (HBO) Therapie wird ebenfalls kontroversiell diskutiert und kann daher in Speziallfälen zur Anwendung kommen. Eine Vac Therapie ist bei postoperativen Wunden ebenfalls erwägbar.

### Diabetische Neuro-Osteoarthopathie – Charcot Fuß

Die diabetische Charcot-Osteoarthopathie (DNOAP: diabetische Neuro-Osteoarthropathie) ist die komplexeste und schwerwiegendste Fußkomplikation. Die Ätiopathogenese ist nicht im Detail geklärt. Die Inzidenz liegt bei 0,3 % pro Jahr.

Ein Charcot Fuß präsentiert sich in der akuten Phase als rot, geschwollen und überwärmt. Es besteht eine mehr oder weniger ausgeprägte Fußdeformität durch das Auftreten von Spontanfrakturen [97, 98]. Zehn bis fünfzehn Prozent der Betroffenen klagen über Schmerzen. In der akuten Phase der Erkrankung besteht immer die Gefahr, dass die Fußdeformität voranschreitet oder es durch das Abkippen von Knochenfragmenten zu chronischen Ulzerationen kommt. Die Amputationsgefahr ist hoch. Die diabetische Neuro-Osteoarthropathie tritt meist im Mittefußbereich auf, kann aber auch an anderen Lokalisationen wie z. B. im Sprunggelenk manifestieren.

Die Diagnose erfolgt primär klinisch. Die Bildgebung wie Röntgen oder MR kann bei der Diagnose zu Problemen führen. Ziel einer Behandlung ist es, den akuten Charcot Fuß in eine chronisch inaktive Form zu überführen. Der chronisch inaktive Charcot Fuß ist wieder belastbar.

Die einzige derzeit anerkannte Behandlungsoption ist eine Druckentlastung für 6–12 Monate, welche im Regelfall mit einem Vollkontaktgips durchgeführt wird. Das Vorliegen eines chronisch inaktiven Charcot Fußes kann angenommen werden, wenn die Rötung und das Ödem sich zurückgebildet hat und der Temperaturunterschied zum nicht betroffenen Fuß unter 2 °C liegt. Der Fuß kann dann mit einem orthopädischen Maßschuh zur Verhinderung von Ulzerationen versorgt werden. Rezidive oder das Auftreten an der kontralateralen Extremität sind keine Seltenheit.

Ein Carcot Fuß sollte immer in einem Zentrum mit entsprechender Expertise versorgt werden.
